# 肾功能受损的初诊多发性骨髓瘤患者应用VRD方案联合自体造血干细胞移植的肾脏反应及预后

**DOI:** 10.3760/cma.j.cn121090-20241211-00564

**Published:** 2025-09

**Authors:** 星玥 吴, 越 黄, 红苗 沈, 红英 尤, 治 严, 妍 谢, 卫芹 姚, 霜 颜, 婧 王, 英颖 翟, 晓兰 施, 京晶 商, 松 金, 灵芝 颜, 德沛 吴, 琤琤 傅

**Affiliations:** 苏州大学附属第一医院血液科，国家血液系统疾病临床医学研究中心，江苏省血液研究所，苏州 215000 National Clinical Research Center for Hematologic Diseases, Jiangsu Institute of Hematology, The First Affiliated Hospital of Soochow University, Suzhou 215006, China

**Keywords:** 多发性骨髓瘤, 肾功能受损, 来那度胺, 自体造血干细胞移植, 肾脏反应, Multiple myeloma, Renal impairment, Lenalidomide, Autologous hematopoietic stem cell transplantation, Renal response

## Abstract

**目的:**

探讨在肾功能受损的多发性骨髓瘤（MM）患者中使用VRD方案联合自体造血干细胞移植（auto-HSCT）的可行性，分析不同肾功能损伤水平的疗效与肾脏反应，探索早期肾脏反应的预后意义及其影响因素。

**方法:**

苏州大学附属第一医院进行的回顾性研究对2018年8月至2022年10月收治的316例初诊多发性骨髓瘤（NDMM）患者按照肾功能分组，分析其临床特征、治疗反应、预后等。计量资料组间比较用*t*检验或秩和检验，计数资料组间比较用卡方检验，生存分析采用Kaplan-Meier法和Log-rank检验，肾脏反应的多因素分析采用Logistic回归。

**结果:**

根据基线估算肾小球滤过率（eGFR）将患者分为正常［≥90 ml·min^−1^·（1.73 m^2^）^−1^，160例］、轻度受损［≥60 ml·min^−1^·（1.73 m^2^）^−1^且<90 ml·min^−1^·（1.73 m^2^）^−1^，55例］、中度受损［≥30 ml·min^−1^·（1.73 m^2^）^−1^且<60 ml·min^−1^·（1.73 m^2^）^−1^，39例］和重度受损［<30 ml·min^−1^·（1.73 m^2^）^−1^，62例］。中或重度受损组患者国际分期系统（ISS）和修订的国际分期系统（R-ISS）分期较晚，血红蛋白水平较低，处于虚弱状态比例更高，轻链和IgD类型的比例更高（均*P*<0.05）。患者的中位年龄和移植率在不同组别间存在差异，与轻度和中度受损组相比，重度受损组患者更年轻（*P*＝0.001），移植率更高（*P*＝0.041）。患者总体客观缓解率（ORR）为93.7％、≥非常好的部分缓解（VGPR）率为82.9％，诱导治疗疗效在各组间差异无统计学意义（均*P*>0.05）。24例（7.6％）确诊时依赖透析，11例（45.8％）诱导治疗期间脱离透析，中位时间为3.0（0.5～4.0）个月。其中有10例患者在透析依赖状态下进行auto-HSCT。89例基线eGFR<50 ml·min^−1^·（1.73 m^2^）^−1^可评估肾脏反应的患者肾脏总体缓解率（RORR）为70.8％（63/89）、肾脏完全应答率为31.5％（28/89）、肾脏部分反应率为11.2％（10/89）、肾脏轻微反应（RMR）率为28.1％（25/89）。有肾脏反应的患者预后较好（总生存：*HR*＝0.36，95％*CI*：0.13～0.99，*P*＝0.049）。尽管中或重度肾功能受损患者在移植过程中出现较多不良反应且造血重建时间延长（*P*<0.05），中位随访33.5（2～65）个月，auto-HSCT仍能显著改善这类患者的预后。多因素分析结果显示1q21+（*OR*＝3.58，95％ *CI*：1.17～11.02，*P*＝0.026）、轻链型（*OR*＝2.86，95％*CI*：1.08～7.69，*P*＝0.036）是肾脏反应不良的独立影响因素。

**结论:**

VRD方案联合auto-HSCT在NDMM（包括肾功能受损）患者的诱导治疗中疗效显著。对于合并肾功能受损的患者，甚至是依赖透析的患者，该方案RORR为70.8％，不良反应可控。达到RMR及以上的患者预后更好，1q21+、轻链型是肾脏反应不良的独立影响因素。

## Abstract (en)

**Objective:**

To investigate the feasibility of the bortezomib, lenalidomide, and dexamethasone（VRD）regimen combined with autologous hematopoietic stem cell transplantation（auto-HSCT）in patients with multiple myeloma（MM）and renal impairment, analyze treatment efficacy and renal responses stratified based on renal dysfunction severity, and explore the prognostic significance of early renal response and its affecting factors.

**Methods:**

This retrospective study, conducted at the First Affiliated Hospital of Soochow University, categorized 316 patients with newly diagnosed MM（NDMM）from August 2018 to October 2022 based on renal function for analysis of clinical characteristics, treatment response, and prognosis. Continuous variables were compared using t-tests or Mann-Whitney U tests, categorical variables utilizing Chi-square tests, survival outcomes employing Kaplan-Meier and Log-rank tests, and renal response predictors with logistic regression.

**Results:**

Patients were stratified based on baseline estimated glomerular filtration rate（eGFR）: normal［≥90 ml·min^−1^·（1.73 m^2^）^−1^, *n*＝160］, mild［≥60 ml·min^−1^·（1.73 m^2^）^−1^ to <90 ml·min^−1^·（1.73 m^2^）^−1^, *n*＝55］, moderate［≥30 ml·min^−1^·（1.73 m^2^）^−1^ to <60 ml·min^−1^·（1.73 m^2^）^−1^, *n*＝39］, and severe impairment［<30 ml·min^−1^·（1.73 m^2^）^−1^, *n*＝62］. Moderate and severe renal impairment correlated with advanced International Staging System/Revised International Staging System classification, lower hemoglobin levels, frailty, and higher light-chain/IgD subtype prevalence（*P*<0.05）. Despite younger age（*P*＝0.001）and higher transplant rates（*P*＝0.041）in severe cases, overall response rates（*ORR*: 93.7％; ≥VGPR: 82.9％）were comparable across groups（*P*>0.05）. Among 24 dialysis-dependent patients at diagnosis, 11（45.8％）achieved dialysis independence after induction［median: 3.0（0.5–4.0）months］, including 10 undergoing auto-HSCT. In 89 evaluable patients［baseline eGFR <50 ml·min^−1^·（1.73 m^2^）^−1^］, renal ORR（RORR）was 70.8％［rapid complete response: 31.5％; rapid partial response: 11.2％; rapid minimal response（RMR）: 28.1％］. Renal response predicted better survival（overall survival: *HR*＝0.36, 95％ *CI*: 0.13–0.99, *P*＝0.049）. Moderate-to-severe renal impairment was associated with increased transplant-related adverse events and delayed engraftment（*P*<0.05）; however, auto-HSCT significantly improved outcomes after 33.5-month median follow-up（range: 2–65 months）. Multivariate analysis identified 1q21+（*OR*＝3.58, 95％ *CI*: 1.17–11.02, *P*＝0.026）and light-chain subtype（*OR*＝2.86, 95％ *CI*: 1.08–7.69, *P*＝0.036）as independent predictors of poor renal response.

**Conclusion:**

VRD regimen plus auto-HSCT demonstrates robust efficacy in NDMM, including patients with renal impairment, with a 70.8％ RORR and manageable toxicity. Achieving ≥RMR correlates with superior prognosis, whereas 1q21+ and light-chain subtype independently predict inferior renal response.

多发性骨髓瘤（MM）是血液系统的恶性肿瘤，其临床特征可被总结为CRAB（高钙血症、肾功能受损、贫血、骨骼破坏）。研究显示有50％以上的MM患者病程中合并肾功能受损，而在初诊患者中由于对肾功能不全定义的不同，占比可达到20％～50％[1]–[2]。其中有2％～12％患者处于透析依赖状态。

基于硼替佐米的诱导治疗被国际骨髓瘤工作组（IMWG）共识评为A级推荐，可作为骨髓瘤相关肾功能受损患者的初始治疗[3]。来那度胺大部分通过肾脏代谢[4]，其在肾功能受损MM患者中的应用因剂量调整而存在争议[5]–[6]。

有研究表明肾功能受损MM患者的肾脏反应可能与生存率的提高有关[7]，而肾脏反应潜在预测因素尚未明确定义。自体造血干细胞移植（auto-HSCT）自问世以来，一直被认为是MM治疗方案中重要的一环，随着医学的进步，其适用范围进一步扩大，包括肾功能受损甚至依赖透析的MM患者[8]。

为了探讨在肾功能受损患者中使用VRD（硼替佐米+来那度胺+地塞米松）方案联合auto-HSCT的可行性，本文旨在一项初诊多发性骨髓瘤（NDMM）应用VRD方案的回顾性研究中，根据不同肾功能受损水平分析疗效与肾脏反应，并探讨早期肾脏反应的预后意义及其影响因素。

## 病例与方法

一、病例

本研究为回顾性研究。对2018年8月至2022年10月苏州大学附属第一医院收治的316例NDMM患者根据肾功能受损程度进行分组分析。MM的诊断、分期、治疗反应评估和风险分层系统参照IMWG指南[9]。本研究中，可以接受移植的患者在接受4个疗程的VRD方案诱导治疗后进行auto-HSCT，随后接受2个疗程的VRD方案巩固治疗。化疗以21 d为1个周期，具体如下：第1、4、8、11天皮下注射硼替佐米1.3 mg/m^2^；第1至14天口服来那度胺25 mg/d（或根据肾功能调整剂量为10 mg/d）；第1、2、4、5、8、9、11、12天静脉注射地塞米松20 mg。标准风险患者接受来那度胺口服维持治疗，直至疾病进展。高危患者［定义为t（4;14）、t（14;16）或del（17p）］接受V+R作为维持治疗，直至疾病进展。纳入患者在诊断时均具有完整临床信息，至少接受2个疗程的VRD方案治疗，并排除非MM导致的急慢性肾功能不全（如糖尿病肾病、药物性肾损害等）。所有患者每次化疗前均进行美国东部肿瘤协作组（ECOG）评分[10]。本研究经苏州大学附属第一医院医学伦理委员会批准（批件号：2024论研批第688号），所有患者均知情同意。

二、来那度胺的剂量调整

血清肌酐（Scr）来自临床病历记录，估算肾小球滤过率（eGFR）根据慢性肾脏病流行病学协作组（CKD-EPI）公式计算[11]：141×min（Scr/κ, 1）^α^×max（Scr/κ, 1）^−1.209^×0.993^Age^×1.018［如为女性］×1.159［如为黑色人种］；其中κ：女性为0.7、男性为0.9；α：女性为−0.329、男性为−0.411；min表示Scr/κ和1相比取较小值，max表示Scr/κ与1相比取较大值。本研究规定患者eGFR≥60 ml·min^−1^·（1.73 m^2^）^−1^，口服来那度胺25 mg/d；30 ml·min^−1^·（1.73 m^2^）^−1^≤eGFR<60 ml·min^−1^·（1.73 m^2^）^−1^，口服来那度胺10 mg/d；eGFR<30 ml·min^−1^·（1.73 m^2^）^−1^或透析依赖，口服来那度胺10 mg，每隔1日用药1次。

三、不良反应和疗效评价

auto-HSCT期间不良反应参照美国常见不良事件评价标准（CTCAE）5.0版。疗效评价依据IMWG评价标准[9]分为严格完全缓解（sCR）、完全缓解（CR）、非常好的部分缓解（VGPR）、部分缓解（PR）、疾病稳定（SD）、疾病进展（PD）。客观缓解率（ORR）为sCR率、CR率、VGPR率、PR率之和。≥VGPR率为sCR率、CR率、VGPR率之和。

四、肾脏疗效

抗骨髓瘤治疗的肾脏反应判断标准参照IMWG的建议[12]。肾脏完全应答（RCR）的定义是基线eGFR从<50 ml·min^−1^·（1.73 m^2^）^−1^增加到≥ 60 ml·min^−1^·（1.73 m^2^）^−1^。肾脏部分反应（RPR）是指eGFR从<15 ml·min^−1^·（1.73 m^2^）^−1^增加到30～59 ml·min^−1^·（1.73 m^2^）^−1^。肾脏轻微反应（RMR）是指基线eGFR从<15 ml·min^−1^·（1.73 m^2^）^−1^改善到15～29 ml·min^−1^·（1.73 m^2^）^−1^，如果基线eGFR为15～29 ml·min^−1^·（1.73 m^2^）^−1^，则改善到30～59 ml·min^−1^·（1.73 m^2^）^−1^。未达到以上标准为肾脏疾病稳定（RSD）。肾脏总体缓解率（RORR）为RCR率、RPR率、RMR率之和。

五、造血干细胞采集和移植

造血干细胞采集方案根据疾病状态和患者的选择，如果肌酐清除率≥30 ml/min，则使用环磷酰胺与粒细胞集落刺激因子（G-CSF），如果肌酐清除率<30 ml/min，则单独使用G-CSF，或联合普乐沙福动员。auto-HSCT预处理方案采用白消安加环磷酰胺（BUCY）或美法仑（200 mg/m^2^），根据肌酐清除率调整剂量[13]。

六、随访

随访信息来自住院和门诊病历以及电话随访记录。随访截止日期为2024年3月1日，中位随访时间为33.5（2～65）个月。无进展生存（PFS）期定义为从疾病确诊或移植后开始到PD或因任何原因死亡的时间；总生存（OS）期定义为从疾病确诊开始至因任何原因死亡或末次随访的时间，移植后OS期定义为从移植日期开始至死亡或末次随访时间。

七、统计学处理

统计分析使用R version 4.4.1软件，绘图使用Graphpad prism 9软件。计数资料统计描述以例（％）表示，计量资料统计描述以*x*±*s*、*M*（范围）、*M*（*Q*_1_，*Q*_3_）表示。均值比较采用*t*检验、方差分析或秩和检验，率的比较采用卡方检验或Fisher精确检验。生存分析采用Kaplan-Meier法进行估计，比较采用对数秩检验。所有检验均采用双尾法，*P*<0.05为差异具有统计学意义。

## 结果

一、患者特征

316例患者根据基线eGFR分为正常［≥90 ml·min^−1^·（1.73 m^2^）^−1^，160例］、轻度受损［≥60 ml·min^−1^·（1.73 m^2^）^−1^且<90 ml·min^−1^·（1.73 m^2^）^−1^，55例］、中度受损［≥30 ml·min^−1^·（1.73 m^2^）^−1^且<60 ml·min^−1^·（1.73 m^2^）^−1^，39例］和重度受损［<30 ml·min^−1^·（1.73 m^2^）^−1^，62例］亚组。所有患者的中位年龄为59（29～73）岁，其中174例（55.06％）为男性。中或重度受损组相较于轻度受损或正常组ECOG评分和查尔斯合并症指数（CCI）得分较高，100％处于虚弱状态，轻链和IgD类型的比例占50％以上，国际分期系统（ISS）和修订的ISS（R-ISS）分期较晚，血红蛋白水平较低，更易合并高钙血症，β_2_-MG水平更高，24h尿蛋白定量更高（*P*<0.05）。但高危细胞遗传学异常比例在4个亚组之间差异无统计学意义（*P*>0.05）。与有肾功能损伤患者相比，无肾功能损伤患者相对更年轻，移植率也更高（均*P*>0.05）。NDMM患者的临床特征见表1。

**表1:** 316例初诊多发性骨髓瘤患者的临床特征

特征	总体（316例）	正常组（160例）	轻度受损组（55例）	中度受损组（39例）	重度受损组（62例）	统计量	*P*值
男性［例（％）］	174（55.1）	85（53.1）	30（54.5）	21（53.8）	38（61.3）	*χ*^2^＝1.244	0.743
年龄［岁，*M*（*Q*_1_, *Q*_3_）］	59.0（53.0，64.0）	57.0（52.0，62.0）	62.0（54.0，66.0）	63.0（56.5，66.5）	58.5（52.0，64.0）	*H*＝17.042	0.001
ECOG评分≥2分［例（％）］	75（23.7）	28（17.5）	14（25.5）	12（30.8）	21（33.9）	*χ*^2^＝8.111	0.044
CCI得分［例（％）］						*χ*^2^＝255.820	<0.001
0分	156（49.4）	114（71.2）	42（76.4）	0（0）	0（0）		
1分	43（13.6）	36（22.5）	7（12.7）	0（0）	0（0）		
≥2分	117（37.0）	10（6.3）	6（10.9）	39（100）	62（100）		
虚弱［例（％）］	155（49.1）	35（21.9）	19（34.5）	39（100）	62（100）	*χ*^2^＝156.820	<0.001
分型［例（％）］						*χ*^2^＝36.020	0.002
IgG型	128（40.5）	67（41.9）	29（52.7）	11（28.2）	21（33.9）		
IgA型	45（14.2）	30（18.8）	7（12.7）	6（15.4）	2（3.2）		
IgD型	8（2.5）	1（0.6）	0（0）	1（2.6）	6（9.7）		
轻链型	115（36.4）	51（31.9）	15（27.3）	20（51.3）	29（46.8）		
寡分泌型	14（4.4）	7（4.4）	3（5.5）	1（2.6）	3（4.8）		
不明	6（1.9）	4（2.5）	1（1.8）	0（0）	1（1.6）		
DS分期［例（％）］						*χ*^2^＝257.170	<0.001
Ⅰ～Ⅱ期	20（6.3）	12（7.5）	3（5.5）	2（5.1）	3（4.8）		
ⅢA期	213（67.4）	148（92.5）	52（94.5）	13（33.3）	0（0）		
ⅢB期	83（26.3）	0（0）	0（0）	24（61.5）	59（95.2）		
ISS分期［例（％）］						*χ*^2^＝151.730	<0.001
Ⅰ期	62（19.6）	51（31.9）	10（18.2）	1（2.6）	0（0）		
Ⅱ期	134（42.4）	93（58.1）	25（45.5）	12（30.8）	4（6.5）		
Ⅲ期	120（38.0）	16（10.0）	20（36.4）	26（66.7）	58（93.5）		
R-ISS分期［例（％）］						*χ*^2^＝76.275	<0.001
Ⅰ期	45（14.2）	37（23.1）	7（12.7）	1（2.6）	0（0）		
Ⅱ期	210（66.5）	117（73.1）	37（67.3）	23（59.0）	33（53.2）		
Ⅲ期	61（19.3）	6（3.8）	11（20.0）	15（38.5）	29（46.8）		
1q21［例（％）］						*χ*^2^＝6.485	0.371
阴性	149（47.2）	82（51.2）	26（47.3）	20（51.3）	21（33.9）		
获得	121（38.3）	55（34.4）	22（40.0）	15（38.5）	29（46.8）		
扩增	46（14.6）	23（14.4）	7（12.7）	4（10.3）	12（19.4）		
TP53［例（％）］	43（13.6）	18（11.2）	10（18.2）	5（12.8）	10（16.1）	*χ*^2^＝2.091	0.554
t（4;14）［例（％）］	63（19.9）	29（18.1）	11（20.0）	11（28.2）	12（19.4）	*χ*^2^＝2.013	0.570
t（14;16）［例（％）］	3（0.9）	1（0.6）	0（0）	0（0）	2（3.2）	*χ*^2^＝4.497	0.213
t（11;14）［例（％）］	35（11.1）	19（11.9）	6（10.9）	4（10.3）	6（9.7）	*χ*^2^＝0.255	0.968
HGB［g/L，*M*（*Q*_1_, *Q*_3_）］	90.00（71.00，109.00）	99.00（77.50，118.00）	90.00（69.50，107.00）	84.00（66.00，100.00）	72.00（63.25，87.75）	*H*＝47.647	<0.001
PLT［×10^9^/L，*M*（*Q*_1_, *Q*_3_）］	165.00（129.50，211.25）	173.50（138.75，216.50）	162.00（141.00，210.50）	155.00（110.50，213.00）	145.00（117.25，187.50）	*H*＝7.445	0.059
钙离子浓度［mmol/L，*M*（*Q*_1_, *Q*_3_）］	2.28（2.15，2.44）	2.24（2.14，2.36）	2.32（2.14，2.48）	2.31（2.25，2.42）	2.34（2.20，2.55）	*H*＝15.871	0.001
白蛋白（g/L, *x*±*s*）	34.87（7.29）	35.26（7.20）	34.21（7.29）	34.66（8.67）	34.57（6.67）	*F*＝0.489	0.791
校正钙离子浓度^a^［>2.75 mmol/L，例（％）］	29（9.2）	6（3.8）	4（7.3）	6（15.4）	13（21.0）	*χ*^2^＝18.037	<0.001
β_2_-MG［mg/L，*M*（*Q*_1_, *Q*_3_）］	4.16（2.49，7.40）	2.80（2.03，3.89）	4.69（3.12，5.81）	6.36（5.00，7.89）	12.85（9.38，19.49）	*H*＝172.970	<0.001
24 h尿蛋白［g/24 h，*M*（*Q*_1_, *Q*_3_）］	1.00（0.23，3.25）	0.35（0.16，1.79）	0.74（0.18，1.65）	3.24（1.38，6.42）	2.22（1.28，4.11）	*H*＝63.394	<0.001
auto-HSCT［例（％）］	246（77.8）	134（83.8）	41（74.5）	25（64.1）	46（74.2）	*χ*^2^＝8.333	0.040

**注** 表中比较均为多组间比较；ECOG：美国东部肿瘤协作组；CCI：查尔森合并症指数；β_2_-MG：β_2_微球蛋白；auto-HSCT：自体造血干细胞移植；^a^校正钙离子浓度（mmol/L）＝钙离子浓度（mmol/L）+0.02［40-白蛋白（g/L）］[14]

二、诱导治疗反应

1. 血液学疗效：经过4个疗程的VRD方案诱导治疗，患者总体ORR为93.7％，≥VGPR率为82.9％。4个亚组间差异无统计学意义（*P*>0.05），见表2。重度肾功能受损患者ORR达到90.3％，49例（79.0％）患者达到了VGPR以上疗效。多参数流式细胞术微小残留病（MRD）检测（10^−4^水平）在不同肾功能受损患者中差异无统计学意义（*P*>0.05）。

**表2:** 初诊多发性骨髓瘤患者不同肾功能受损组的诱导血液学反应［例（％）］

疗效情况	患者总体（316例）	正常组（160例）	轻度受损组（55例）	中度受损组（39例）	重度受损组（62例）
治疗反应					
sCR	36（11.4）	15（9.4）	9（16.4）	5（12.8）	7（11.3）
CR	102（32.3）	55（34.4）	17（30.9）	12（30.8）	18（29.0）
VGPR	124（39.2）	63（39.4）	21（38.2）	16（41.0）	24（38.7）
PR	34（10.8）	19（11.9）	7（12.7）	1（2.6）	7（11.3）
SD或PD	12（3.8）	6（3.8）	0（0）	2（5.1）	4（6.5）
不明	8（2.5）	2（1.2）	1（1.8）	3（7.7）	2（3.2）
客观缓解	296（93.7）	152（95.0）	54（98.2）	34（87.2）	56（90.3）
≥VGPR	262（82.9）	133（83.1）	47（85.5）	33（84.6）	49（79.0）
MRD情况					
阴性	131（41.5）	68（42.5）	21（38.2）	19（48.7）	23（37.1）
阳性	161（50.9）	84（52.5）	28（50.9）	15（38.5）	34（54.8）
不明	24（7.6）	8（5.0）	6（10.9）	5（12.8）	5（8.1）

**注** sCR：严格意义的完全缓解；CR：完全缓解；VGPR：非常好的部分缓解；PR：部分缓解；SD：疾病稳定；PD：疾病进展；客观缓解：sCR+CR+VGPR+PR；MRD：微小残留病

2. 肾脏反应：89例基线eGFR<50 ml·min^−1^·（1.73 m^2^）^−1^的患者可评估肾脏反应，RORR为70.8％（63/89），其中RCR率为31.5％（28/89）、RPR率为11.2％（10/89）、RMR率为28.1％（25/89）。重度肾功能受损患者的肾脏反应如下，RORR为71.0％（44/62），其中，RCR率为21.0％（13/62）、RPR率为16.1％（10/62）、RMR率为33.9％（21/62）。24例（7.6％）患者在确诊时依赖透析，其中11例（45.8％）患者在诱导期间脱离透析，中位时间为3.0（0.5～4.0）个月。

三、造血干细胞采集和auto-HSCT

诱导治疗后共有246例患者接受了auto-HSCT，其中6例进行了挽救性移植。患者从确诊到接受auto-HSCT的中位时间为6.9（3.9～16.9）个月。其中40例患者在移植时尚未获得RCR，10例患者在透析依赖状态下进行auto-HSCT。采集CD34^+^细胞数量中位数为4.92（0.83～14.34）×10^6^/kg。肾功能中或重度受损的患者为40例，正常或轻度受损的患者为206例，两组人群采集的CD34^+^细胞数量中位数分别为2.66（0.83～10.39）×10^6^/kg和5.48（0.99～14.34）×10^6^/kg，*P*<0.001。患者总体移植时输注CD34^+^细胞数量中位数为3.71（0.83～13.28）×10^6^/kg，肾功能中或重度受损和正常或轻度受损的患者输注的CD34^+^细胞数量中位数分别为2.59（0.83～10.39）×10^6^/kg和3.93（0.99～13.28）×10^6^/kg，*P*<0.001。进行auto-HSCT时，患者总体住院时间中位数为22（18～45）d，肾功能中或重度受损和正常或轻度受损的患者住院的中位时间分别为24（18～45）d和22（18～34）d，*P*<0.001。

移植时所有患者均出现4级的中性粒细胞减少和血小板减少，粒系造血重建的中位时间为11（7～16）d，脱离血小板输注的中位时间为12（0～28）d。肾功能中或重度受损和正常或轻度受损的患者粒系造血重建的中位时间分别为12（8～15）d和10（7～16）d。两个亚组脱离血小板输注中位时间分别为11（0～28）d和14（10～25）d。肾功能中或重度受损患者粒系造血重建和脱离血小板输注的中位时间更长（均*P*<0.001）。

除血液学不良反应，其他移植不良事件见表3。95％以上的患者出现不同程度的胃肠道反应和不同部位的黏膜炎表现。其他比较常见的有转氨酶升高（30.9％）、中性粒细胞减少性发热（28.5％）和肌酐升高（18.7％）。统计结果显示，肾功能中或重度受损组发生肌酐升高、心血管事件和其他不良事件较多（*P*<0.05）。其他不良事件中，肾功能中或重度受损组3例发生肿瘤溶解综合征，3例发生植入综合征，其中2例需进入ICU治疗；肾功能正常或轻度受损组3例发生植入综合征、1例发生败血症、1例发生肿瘤溶解综合征、1例结膜出血伴隆起、1例支气管扩张伴咯血、1例血栓形成。尽管如此，患者不良反应均可控，移植相关死亡率为0。

**表3:** 初诊多发性骨髓瘤患者自体造血干细胞移植相关不良事件［例（％）］

不良事件	总体（246例）	正常或轻度受损组（206例）	中或重度受损组（40例）	*χ*^2^值	*P*值
恶心	238（96.7）	200（97.1）	38（95.0）	0.467	0.493
呕吐	147（59.8）	126（61.2）	21（52.5）	1.119	0.333
腹泻	196（79.7）	164（79.6）	32（80.0）	0.006	0.939
中性粒细胞减少性发热	70（28.5）	54（26.2）	16（40.0）	3.110	0.080
消化道出血	13（5.3）	10（4.9）	3（7.5）	0.473	0.491
黏膜炎	244（99.1）	204（99.0）	40（100）	–	0.726
转氨酶升高	76（30.9）	67（32.5）	9（22.5）	1.590	0.218
肌酐升高	46（18.7）	22（10.7）	24（60.0）	65.585	<0.001
心血管事件	10（4.1）	6（2.9）	4（10.0）	4.315	0.028
过敏或输注反应	18（7.3）	17（8.3）	1（2.5）	1.638	0.201
其他	14（5.7）	8（3.9）	6（15.0）	9.550	0.002

**注** –：Fisher精确检验不计算统计量

四、生存结果

截至2024年3月1日，中位随访时间为33.5（2～65）个月，316例患者总体中位PFS期预计为55［95％*CI*：49～无法获得（NA）］个月，而中位OS期尚未达到，60个月的累积OS率为73.1％（95％*CI*：58.6％～82.5％）。

根据肾功能分层的4组患者PFS和OS比较如图1A、B所示，可见随着肾功能受损程度的加重，患者的PFS和OS期缩短。而在重度肾功能受损患者中，需要依赖透析的患者具有更差的预后［中位PFS期：37（95％*CI*：20～NA）个月对55（95％*CI*：38～NA）个月，*P*＝0.097；中位OS期：45（95％*CI*：38～NA）个月对未达到，*P*＝0.012］。eGFR为60 ml·min^−1^·（1.73 m^2^）^−1^以下和以上患者中位PFS期分别为52（95％*CI*：36～NA）个月和未达到（*HR*＝1.57，95％*CI*：1.04～2.37，*P*＝0.020），60个月累积OS率为81.8％（95％*CI*：70.4％～89.1％）和55.1％（95％*CI*：26.7％～76.4％）（*HR*＝2.41，95％ *CI*：1.29～4.48，*P*＝0.002），差异具有统计学意义（图1C、D）。

**图1:**
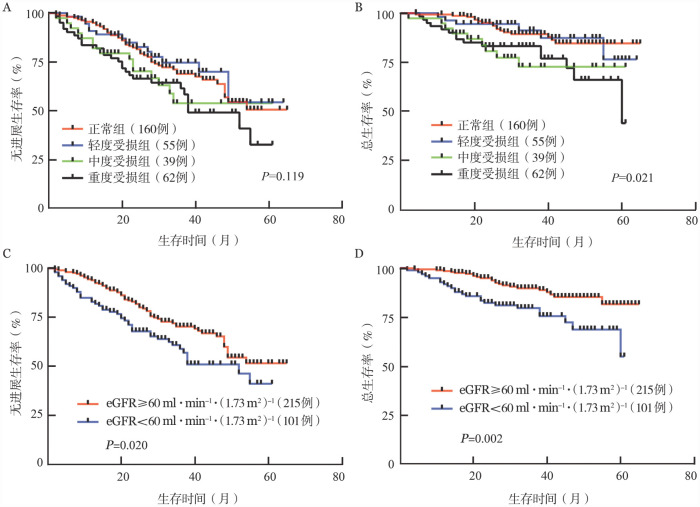
316例初诊多发性骨髓瘤患者的无进展生存（PFS）和总生存（OS）曲线 **A、B** 不同肾功能受损程度患者的PFS和OS曲线；**C、D** 以eGFR为60 ml·min^−1^·（1.73 m^2^）^−1^为界的PFS和OS曲线

对于89例基线eGFR<50 ml·min^−1^·（1.73 m^2^）^−1^可评估肾脏反应的患者，与肾脏无反应患者（26例）相比，肾脏有反应（≥RMR）的患者（63例）预后较好，中位PFS期为55（95％*CI*：37～NA）个月对38（95％*CI*：20～NA）个月，中位OS期为未达到对45（95％*CI*：38～NA）个月（PFS：*HR*＝0.65，95％*CI*：0.31～1.39，*P*＝0.271；OS：*HR*＝0.36，95％*CI*：0.13～0.99，*P*＝0.049）。而对于需要透析治疗的患者，不管在诱导治疗期间能否脱离透析，在预后方面差异无统计学意义（*P*>0.05）。同时我们比较肾功能受损患者与正常患者在诱导治疗期间的恢复程度，在至少达到RMR的疗效下，二者PFS和OS期差异无统计学意义（*P*>0.05）。提示患者在诱导治疗期间达到RMR，预后可得到改善。

如图2A、B所示，auto-HSCT可改善NDMM患者的预后［未移植对移植：中位PFS期：30（95％*CI*：23～NA）个月对未达到，*P*<0.001；中位OS期：未达到对未达到，*P*<0.001］。在各个肾功能受损程度分组（包括初诊时依赖透析治疗和诱导期肾脏反应已达到RMR的患者）中均观察到auto-HSCT有助于患者PFS和OS期的延长。然而，中或重度肾功能受损患者（40例）auto-HSCT后的PFS和OS期短于肾功能正常或轻度受损组［中位PFS期：31（95％*CI*：30～NA）个月对未达到，*P*＝0.022；中位OS期：52（95％*CI*：42～NA）个月对未达到，*P*＝0.008；图2C、D］。移植后3个月，57例患者可继续评估肾脏反应，RCR 23例（40.4％），RPR 9例（15.8％），RMR 17例（29.8％），RSD 8例（14.0％）。2例由移植前RMR改善至RCR，2例由RSD改善至RMR，1例由RMR改善至RPR。

**图2:**
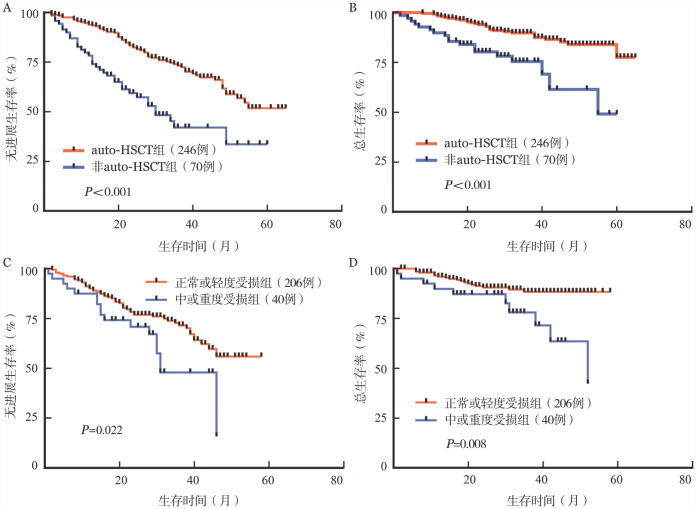
初诊多发性骨髓瘤患者自体造血干细胞移植（auto-HSCT）相关的无进展生存（PFS）和总生存（OS）曲线 **A、B** auto-HSCT组与非auto-HSCT患者的PFS和OS比较；**C、D** 正常或轻度受损组和中或重度受损组患者auto-HSCT后PFS和OS比较

五、诱导期肾脏反应的Logistic回归分析

以89例基线eGFR<50 ml·min^−1^·（1.73 m^2^）^−1^可评估肾脏反应患者是否能达到RMR为分组条件，比较两组人群的疾病特征，肾脏反应不良组1q21+比例较高，HGB、PLT以及β_2_-MG水平更高（均*P*<0.05）。以是否能达到至少RMR的肾脏学疗效为结局变量，单因素分析显示轻链型（*OR*＝2.70，95％ *CI*：1.04～7.14，*P*＝0.042）、1q21+（*OR*＝3.36，95％ *CI*：1.12～10.04，*P*＝0.030）为肾脏反应不良的预测因素。多因素分析结果显示，轻链型（*OR*＝2.86，95％*CI*：1.08～7.69，*P*＝0.036）、1q21+（*OR*＝3.58，95％ *CI*：1.17～11.02，*P*＝0.026）为肾脏反应不良的独立影响因素。

## 讨论

本研究数据显示，初诊肾功能受损患者预后劣于肾功能正常患者，但VRD方案诱导治疗后达到至少RMR的患者与肾功能正常患者相比具有相似的PFS和OS。肾功能早期恢复不仅能降低慢性肾脏病进展风险，还能改善治疗耐受性、改善疗效和生活质量、减少感染等合并症的发生，从而延长生存期。

专门针对肾功能受损患者的临床研究较少。本研究中，VRD方案治疗NDMM患者血液学疗效ORR达到93.7％、≥VGPR率为82.9％、≥CR率为43.7％，在肾功能受损程度分层亚组中差异无统计学意义。本研究重点关注肾功能受损患者的肾脏反应。Bachmann等[15]研究结果显示，VRD、VCD、RAD方案的肾脏反应差异无统计学意义，RORR分别为52％、68％、63％。一项针对133例NDMM［eGFR<60 ml·min^−1^·（1.73 m^2^）^−1^］患者的研究显示，81％的硼替佐米组患者、74％的沙利度胺组患者以及61％的来那度胺组患者肾功能改善（即至少达到RMR，*P*＝0.153）[16]。FIRST试验中，Rd和MPT方案治疗的肾脏CR率分别为23.8％和14.3％，肾脏PR率分别为11.1％和7.7％[17]。一项来自中国的多中心前瞻性研究结果显示，PVD方案在肾功能受损［定义为eGFR<40 ml·min^−1^·（1.73 m^2^）^−1^］的NDMM患者中有较高的RORR，治疗3个月时，46例患者（75.4％）达到肾脏反应，RCR率为29.5％、RPR率为9.8％、RMR率为36.1％[6]。另有一项在PVD方案基础上加用塞利尼索用于NDMM肾损伤患者的研究正在进行。本研究VRD方案诱导治疗的RORR达到70.8％，重度肾功能受损患者ORR也能达到71.0％。以硼替佐米为基础的三药联合方案已成为NDMM患者的标准诱导方案，虽然来那度胺的用量需根据肌酐清除率进行调整，但在骨髓瘤缓解和肾脏缓解上具有良好的疗效和安全性。

以达雷妥尤单抗（Dara）为代表的单抗药物不通过肾脏代谢，因此，其在肾功能不全患者中的使用成为新的研究热点。但包含Dara的MAIA[18]、GRIFFIN[19]、MASTER[20]、Hovon-143[21]等研究中均排除了肾功能受损严重患者，关于单抗药物治疗NDMM患者的肾脏反应数据少。一项硼替佐米、Isatuximab、环磷酰胺和地塞米松方案治疗符合移植条件的肾功能受损MM患者的临床试验（NCT04240054）正在招募阶段，一项Dara皮下注射制剂联合环磷酰胺、硼替佐米、地塞米松治疗合并肾衰竭NDMM患者的临床试验（NCT06142396）正在注册阶段。

肾脏恢复不良预示伴有肾功能受损的MM患者的生存率低。在一项使用高通量透析器的临床试验中，化疗第1个周期结束时血清游离轻链浓度为<500 mg/L与更好的肾脏反应相关，该值被认为是管型形成的阈值，而基线游离血清轻链水平不具有预测性[22]。Hutchison等[23]的研究表明肾脏反应与血清游离轻链降低之间呈线性关系，没有绝对的血清游离轻链降低阈值，但在治疗第21天时，游离轻链降低60％与80％患者的肾脏反应相关。本研究多因素回归分析结果显示，1q21+、轻链型是患者诱导期肾脏反应不良的独立影响因素。本研究显示合并肾功能受损的MM患者更易呈现轻链型，而非常见的IgG型，轻链的相对分子质量约为22×10^3^，远小于完整的Ig（IgG的相对分子质量约为150×10^3^），轻链更易通过肾小球滤过膜进入肾小管，形成轻链管型肾病，其为MM合并肾损伤的常见病因[24]。与文献报道[25]一致，单纯轻链型MM患者（*HR*＝2.841，95％ *CI*：1.471～5.486，*P*＝0.002）是肾脏反应不良的独立预测因素。而1q21获得或扩增已被多项指南纳入高危细胞遗传学异常，二次修订的国际分期系统（R2-ISS）也将1q纳入预后分层模型[26]，1q21+患者肾功能不易恢复的根本原因有待大型数据的考证和机制实验的确认。

auto-HSCT在MM患者整体治疗方案中仍处于不可撼动的地位，包括肾功能受损患者[3],[8],[27]。本研究观察到患者行auto-HSCT的获益，包括依赖透析患者相关死亡率为0。尽管如此，肾功能受损患者的干细胞采集和输注数量较少，移植时骨髓抑制恢复更慢，出现不良反应更多，住院时间更长。因此，auto-HSCT需要在经验丰富的治疗中心进行，并加强个性化管理。然而，本研究仍存在诸多局限，样本量有限，未系统分析游离轻链动态变化与肾脏反应的关系，随访时间尚短等。而新的联合CD38单抗的联合方案正在进行中，以克服肾功能损伤相关的预后不良。
